# Improving the representation of peptide-like inhibitor and antibiotic molecules in the Protein Data Bank

**DOI:** 10.1002/bip.22434

**Published:** 2014-03-25

**Authors:** Shuchismita Dutta, Dimitris Dimitropoulos, Zukang Feng, Irina Persikova, Sanchayita Sen, Chenghua Shao, John Westbrook, Jasmine Young, Marina A Zhuravleva, Gerard J Kleywegt, Helen M Berman

**Affiliations:** 1 RCSB Protein Data Bank, Department of Chemistry and Chemical Biology, Rutgers, The State University of New JerseyPiscataway, NJ, 08854-8076; 2 RCSB Protein Data Bank, San Diego Supercomputer Center and Skaggs School of Pharmacy and Pharmaceutical Sciences, University of California, San DiegoLa Jolla, CA, 92093-0537; 3 Protein Data Bank in Europe (PDBe), European Molecular Biology Laboratory, European Bioinformatics Institute, Wellcome Trust Genome CampusHinxton, Cambridge, CB10 1SD, UK

**Keywords:** peptide-like inhibitor, peptide-like antibiotic, Protein Data Bank

## Abstract

With the accumulation of a large number and variety of molecules in the Protein Data Bank (PDB) comes the need on occasion to review and improve their representation. The Worldwide PDB (wwPDB) partners have periodically updated various aspects of structural data representation to improve the integrity and consistency of the archive. The remediation effort described here was focused on improving the representation of peptide-like inhibitor and antibiotic molecules so that they can be easily identified and analyzed. Peptide-like inhibitors or antibiotics were identified in over 1000 PDB entries, systematically reviewed and represented either as peptides with polymer sequence or as single components. For the majority of the single-component molecules, their peptide-like composition was captured in a new representation, called the subcomponent sequence. A novel concept called “group” was developed for representing complex peptide-like antibiotics and inhibitors that are composed of multiple polymer and nonpolymer components. In addition, a reference dictionary was developed with detailed information about these peptide-like molecules to aid in their annotation, identification and analysis. Based on the experience gained in this remediation, guidelines, procedures, and tools were developed to annotate new depositions containing peptide-like inhibitors and antibiotics accurately and consistently. © 2013 Wiley Periodicals, Inc. Biopolymers 101: 659–668, 2014.

## INTRODUCTION

The Protein Data Bank (PDB) is the single global archive of three-dimensional (3D) structural data of biological macromolecules and their complexes. It is managed by the Worldwide PDB (wwPDB; http://wwpdb.org;^1^ a collaborative organization with four partners—the Research Collaboratory for Structural Bioinformatics (RCSB PDB; http://rcsb.org), the PDB in Europe (PDBe; http://pdbe.org), the PDB Japan (PDBj; http://pdbj.org), and the Biological Magnetic Resonance Data Bank (BMRB; http://bmrb.wisc.edu). The partners act as deposition, processing, and distribution centers for PDB data. They collaborate on developing annotation procedures and guidelines, data representation models and formats, and work with community experts to define data quality and validation standards.^2^ Occasionally, the wwPDB undertakes large-scale remediation efforts to improve the data representation, consistency, integrity, and usability of the archive. For instance, past archive-wide remediation projects^3^,^4^ have focused on (i) improving the chemical description of the monomer units of the biological polymers and small molecule ligands in the PDB, (ii) standardizing the atom nomenclature to conform to IUPAC recommendations, (iii) updating sequence and taxonomy database references, (iv) improving the representation of viruses, and (v) verifying primary citation assignments.

Although the PDB is primarily a repository for experimentally determined structures of proteins and nucleic acids, a wide variety of other biologically relevant molecules are archived in it, including metals, inorganic ions, cofactors, ligands, substrates, inhibitors, antibiotics, and various drugs. While some of the inhibitor and antibiotic molecules are derived from natural sources, others have been designed for specific purposes. In the PDB, the majority of these diverse biologically interesting molecules are found in complex with proteins or nucleic acid polymers, shedding light on the functions of the target molecules. The structures of some of these molecules have been studied in their isolated form too, for example, antibiotics such as thiostrepton^5^ and vancomycin.^6^ The structure and biosynthesis of these molecules involve a wealth of interesting chemistry, both in the molecules themselves and in their interactions with target macromolecules.

Peptide-like compounds, many of which are pharmaceutically relevant antibiotics or inhibitors of key enzymes in metabolic pathways, form an important subset of the biologically relevant small molecules in the PDB. In the past, these molecules occurred infrequently in PDB entries and were annotated on a case-by-case basis, sometimes resulting in inconsistent representations. Given their importance and the increasing number of structure depositions that include peptide-like inhibitors and antibiotics, a remediation project was carried out. The goal was to make the representation and annotation of peptide-like inhibitors and antibiotics consistent across the PDB archive so as to facilitate their identification, retrieval, comparison and analysis. One important outcome of this work is a new reference dictionary that contains additional annotations for this class of biologically important molecules.

## RESULTS

### Remediation

The first step in remediation was the identification of the peptide-like inhibitor and antibiotic molecules in the PDB archive. This was challenging as some of the peptide-like molecules were represented as large single components, while others were represented as polymers or as a set of residues with explicit linkages between them. In many cases, the list of linkages between the residues was incomplete or incorrect and sometimes the same molecule was represented in different ways in different entries.

Over a thousand PDB entries were found to contain peptide-like inhibitors and antibiotics (∼150 PDB entries with ∼60 different peptide-like antibiotics and ∼850 PDB entries with ∼310 peptide-like inhibitors). Some of these peptide-like inhibitors and antibiotics are modified, ribosomally synthesized gene products, such as thiostrepton (PDB entry 1e9w).^5^ Others are products of nonribosomal enzymatic synthesis, such as vancomycin (PDB entry 1sho).^6^ Finally, some of these compounds were specifically designed and synthesized in vitro, such as the protease inhibitor d-phenylalanyl-l-prolyl-l-arginine chloromethyl ketone or PPACK for short (PDB entry 1a0h).^7^ The representation of the peptide-like molecules was reviewed and, where necessary, modified to ensure that their composition was easily decipherable. Each peptide-like inhibitor or antibiotic was represented consistently and in its entirety, including all linkages required to describe the molecule.

**Figure 1:**
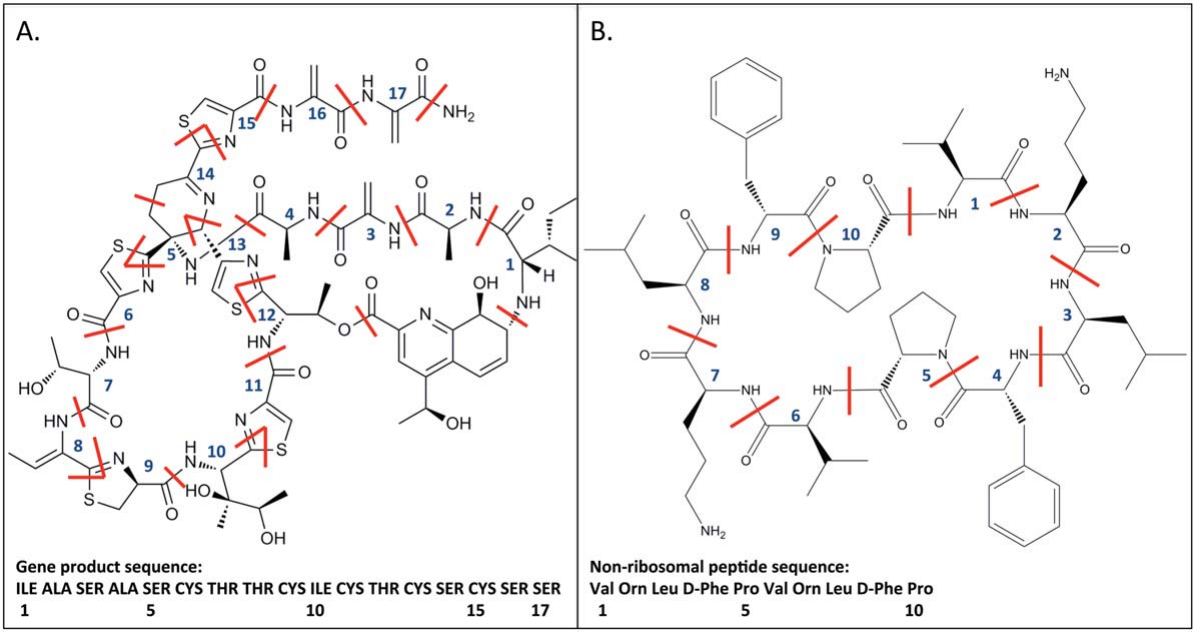
The chemical structures and sequences of (A) thiostrepton (PDB entry 1e9w)^5^ and (B) gramicidin S (PDB entry 1tk2).^8^ The chemical diagrams show cyclizations and modifications that lead to formation of the final antibiotic molecule. Red lines indicate the boundaries of the chemical components in the polymer, while the numbers indicate the correspondence with the gene or nonribosomal product.

Most peptide-like antibiotics (ribosomal and nonribosomal products) contain at least two consecutive peptide bonds and are represented as peptides with polymer sequences. In addition to peptide bonds, many of these molecules contain unusual linkages between their components, for instance, due to the formation of a thiazole ring (as in thiostrepton, PDB entry 1e9w)^5^ (Figure 1A), or the cyclization of the polymer (as in gramicidin S, PDB entry 1tk2)^8^ (Figure 1B). All these special linkages were explicitly defined for all instances in a given PDB entry. The peptide-like inhibitors in ∼370 PDB entries also contain at least two consecutive peptide bonds. Therefore, these were represented with polymer sequences and all nonstandard linkages were explicitly defined.

**Figure 2:**
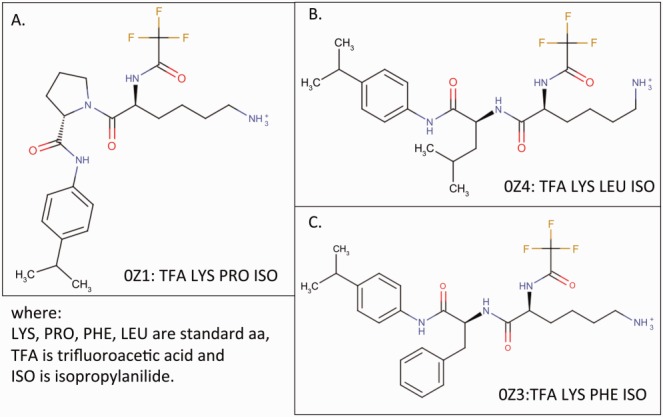
Chemical structure of three trifluoroacetyl-dipeptide-anilide inhibitors of elastase.^9^ In each case, the inhibitor's chemical component name is followed by its subcomponents (following the colon). (A) Chemical structure of inhibitor 0Z1 from PDB entry 1ela; (B) inhibitor 0Z4 from PDB entry 1elb; and (C) inhibitor 0Z3 from PDB entry 1elc.

The peptide-like inhibitors in the remaining (∼480) entries were represented as single components. Many of these single-component inhibitors contain standard or modified amino acids linked via a combination of nonconsecutive peptide bonds and/or nonpeptide linkages. Molecules with fewer than two consecutive peptide bonds are not represented as a polymer sequence. A new representation, called subcomponent sequence, was developed to capture the identities of the standard or modified amino acids, linkers, and other chemical components within these molecules. Similar to any residue in a polymer sequence, all subcomponents are completely defined in the Chemical Component Dictionary (CCD)^3^ maintained by the wwPDB. Where possible, the subcomponent sequence of peptide-like molecules is listed from the amino (N) to the carboxyl (C) end. The subcomponent sequence representation facilitates pseudosequence comparison of the single component peptide-like molecules. For example, three different inhibitors 0Z1, 0Z4, and 0Z3, from PDB entries 1ela, 1elb, and 1elc, respectively,^9^ are shown in Figure 2 along with their subcomponent sequences. The subcomponent following lysine was changed in each of these inhibitors to study its impact on the binding and function of the inhibitor molecule.^9^

**Figure 3:**
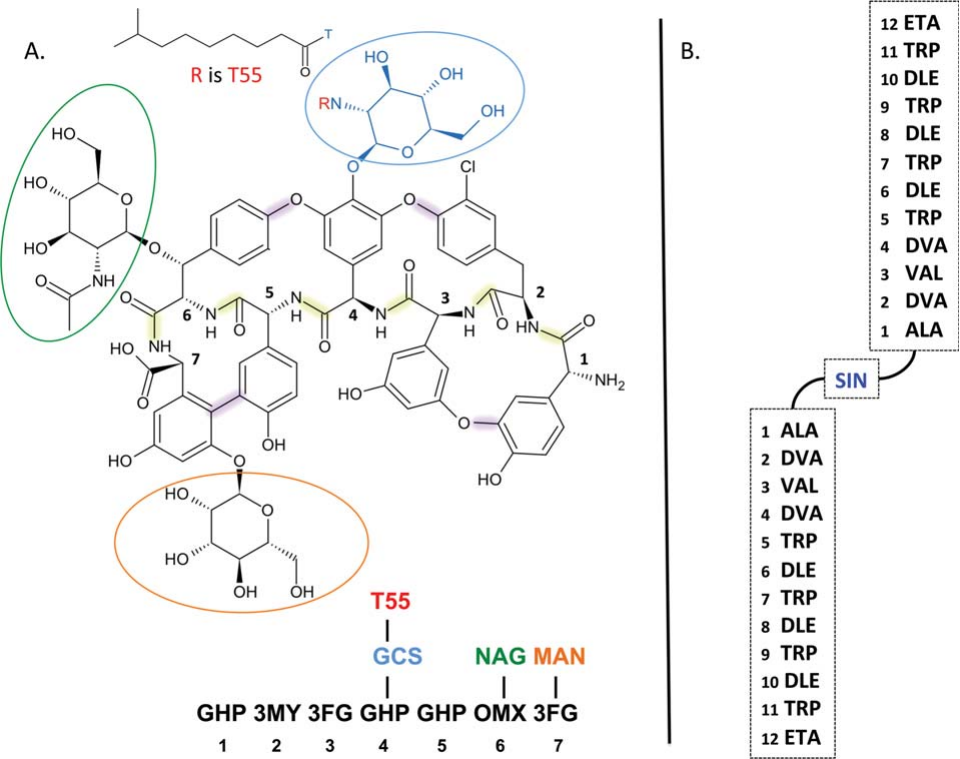
Representation of grouped peptide-like molecules. (A) Example of a peptide-like antibiotic that is a derivative of teicoplanin (from PDB entry 3vfj).^10^ The molecule shown here has lost a single chlorine atom during the experiment and is chemically different from naturally occurring teicoplanin. Both chemical structure and components list show that teicoplanin has a peptide core (shown in black), decorated with three saccharides (circled in blue, green, and orange) and a fatty acid (shown as R in red). The chemical components in the peptide core are numbered from 1 to 7. Bonds highlighted in green denote the peptide linkages between residues in the peptide core, while the purple bonds mark the covalent linkages between side-chains of the peptide residues. (B) Schematic representation of residues in the 22-mer “minigramicidin” (PDB entry 1kqe)^11^ showing two copies of the terminal 11-mer domains of gramicidin A, covalently linked in a head-to-head fashion. The linker between the two molecules is succinic acid (SIN).

Some peptide-like antibiotics are composed of a peptide core (with a polymer sequence) and other polymer or nonpolymer components. For example, the glycopeptide antibiotic teicoplanin is composed of a peptide core, decorated with three monosaccharides and a fatty acid. Figure 3A shows the chemical structure and components of a derivative of teicoplanin found in PDB entry 3vfj.^10^ Currently, the PDB can only accommodate linear sequences of polymers; therefore, a new representation called “group” was developed for such complex compounds. A group includes all polymeric and nonpolymeric constituents of a molecule, along with explicit specifications of the linkages between them. This representation was also used for peptide-like molecules in which the directionality of the peptide linkages is not exclusively from amino to carboxyl terminus (N-to-C), such as in the modified gramicidin in PDB entry 1kqe^11^ (shown in Figure 3B), which is composed of two short peptides linked in a head-to-head manner through a linker moiety.

**Figure 4:**
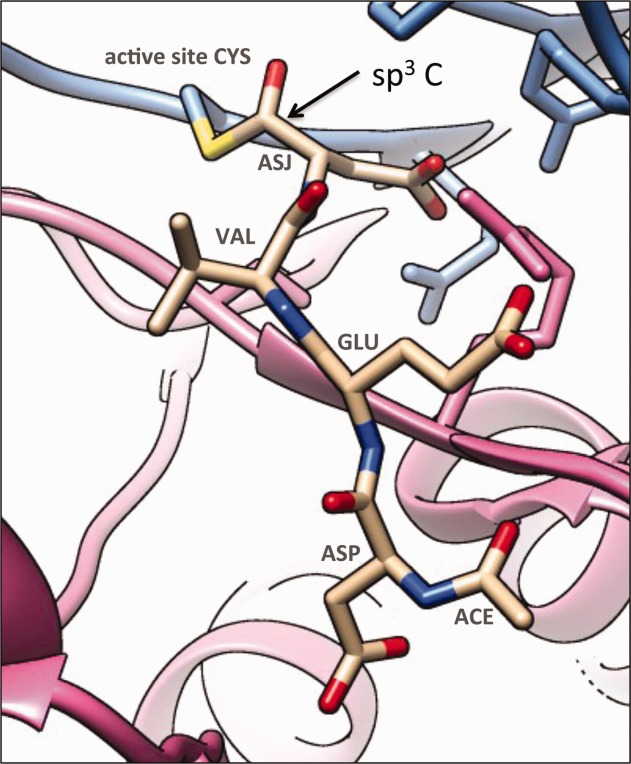
Covalent binding of the peptide-like inhibitor ACE-ASP-GLU-VAL-ASJ to its target, caspase 3, in PDB entry 4dcp.^12^ While the carbonyl carbon (marked with an arrow) of the C-terminal residue in the unbound inhibitor (of type ASA) is sp^2^, its hybridization state in the bound hemithioacetal product is sp^3^ and the residue is of type ASJ.

The binding environment of the peptide-like molecules was explicitly annotated, highlighting all residues in the target macromolecule that participate in covalent and noncovalent interactions. Special attention was given to the chemistry of peptide-like molecules that undergo significant chemical changes upon binding the target molecule. For example, the active site cysteine residue of caspase-3 attacks the carbonyl group of the aspartate aldehyde-based inhibitor Ac-DEVD-Cho to form a covalent thiohemiacetal linkage (PDB entry 4dcp)^12^ (Figure 4). As a result, both the hybridization state and geometry of the linked carbon are different compared with that in the unbound inhibitor. Thus, the aspartate–aldehyde residue used in this inhibitor's polymer sequence is ASJ (instead of the ASA, used for the unbound inhibitor), denoting its different chemical properties. Similarly, bound and unbound forms of single-component, peptide-like inhibitors were annotated as distinct chemical components with different subcomponent sequences to highlight changes in hybridization state.

The different representations of the remediated peptide-like inhibitor and antibiotic molecules and their specific annotations are summarized in TableI. For PDB entries in which the representation of a peptide-like molecule was changed compared with the original released version, a new category of data items was included that provides atom-by-atom mapping of the atom names in the pre-and post-remediation representations. This allows users to map atom names used in the literature to specific residues and atoms in the remediated PDBx^13^ and PDB format^14^ (http://www.wwpdb.org/docs.html) files.

**Table I:** Overview of Representation and Annotation of Polymeric, Single Component and Grouped Peptide-Like Molecules in the PDB

Molecule Properties	Peptide with Polymer Sequence	Single Component with Subcomponents	Group (Peptide Core with Additional Polymer and/or Nonpolymer Components)
Name	Polymer name is listed along with names of other polymers in the PDB entry	Component name is listed along with other ligands, ions or components in the PDB entry	Group name is listed for the complete molecule – including all polymer and nonpolymer components
Source	Source organism is included for naturally derived polymers	Not applicable (n/a) as most molecules are designed	Source organism is included for the polymeric portion(s) of the grouped molecule
Composition and linkage	• Polymer sequence is listed along with other polymers in the PDB entry.	• Apparent “sequence” is described in subcomponent sequence.	• All constituents of the molecule group are defined in PDB entry
	• Standard peptide linkages between component residues are implied	• Explicit linkages between subcomponents are listed in the corresponding PRD files.	• Sequence of polymeric components is described just as any other polymer
	• All nonstandard linkages are explicitly described in the PDB entry and PRD file.	• All linkages between atoms are listed in the CCD	• Linkages between polymeric and nonpolymeric constituents explicitly defined in PDB and PRD entries.
Reference	Sequence database reference for polymer is included, where available	n/a	Sequence database reference for polymeric components is included, where available
Structure	Regular regions of 3D structure (such as helices/sheets) are described, where appropriate	n/a	Regular regions of 3D structure (such as helices/sheets) are described for polymeric components, where appropriate
Binding environment	Residues interacting with or surrounding the polymer are highlighted	Residues interacting with or surrounding the component are highlighted	Residues interacting with or surrounding the grouped molecule are highlighted
Function	Overall function of the polymer is described	Overall function of the component is described	Overall function of the grouped molecule is described

### Biologically Interesting Molecule Reference Dictionary

The large body of chemical, structural, and functional information, gathered during the remediation of the peptide-like inhibitors and antibiotics, was organized to create a new reference dictionary named Biologically Interesting molecule Reference Dictionary (BIRD). This dictionary comprises Peptide-like molecules Reference Dictionary (PRD) entries with unique identifiers and detailed descriptions for each chemically distinct peptide-like inhibitor or antibiotic molecule. The entries include information about the composition, connectivity, chemical structure description, and functions of these molecules (TableII). Remediated and newly deposited PDB entries containing peptide-like inhibitors and antibiotics now include PRD identifiers in the PDBx format files. The corresponding PRD files are available for download from the wwPDB ftp server (http://ftp://ftp.wwpdb.org/pub/pdb/data/bird/prd/) and its mirrors at the wwPDB partner sites.

**Table II:** Overview of Information Content of PRD and FAM Entries in the BIRD Resource

Property	PRD File	FAM File
Identifier	PRD_######, e.g., PRD_000001	FAM_######, e.g., FAM_000001
Name	Molecule name	Family name
Description	• Molecular formula and weight	• List of member PRD identifiers
	• Specific function (class)	• Literature references
	• Structural details (type)	
Chemical details	• List of polymer and nonpolymer entities comprising the molecule	• Synonyms for PRD family members collected from various resources including the PDB and primary citations
	• Polymer or subcomponent sequence• Description of how all components in the molecule are linked	• Reference database IDs and links to various resources that provide information about the family members
	○ Intra-entity linkages between components in polymer entities	• Family specific annotations
	○ Inter-entity linkages between polymer segments and nonpolymer components (for molecules with grouped representation)	○ IDs and information about related small molecule crystal structures in the CSD^16^
		○ Corresponding literature references
Biological details	Name of organism producing the molecule (for naturally produced molecules) and source of this information (e.g. from a database, author or literature)	Annotations about structural, functional and mechanistic details for family members, including pharmacological action (where appropriate)

**Figure 5:**
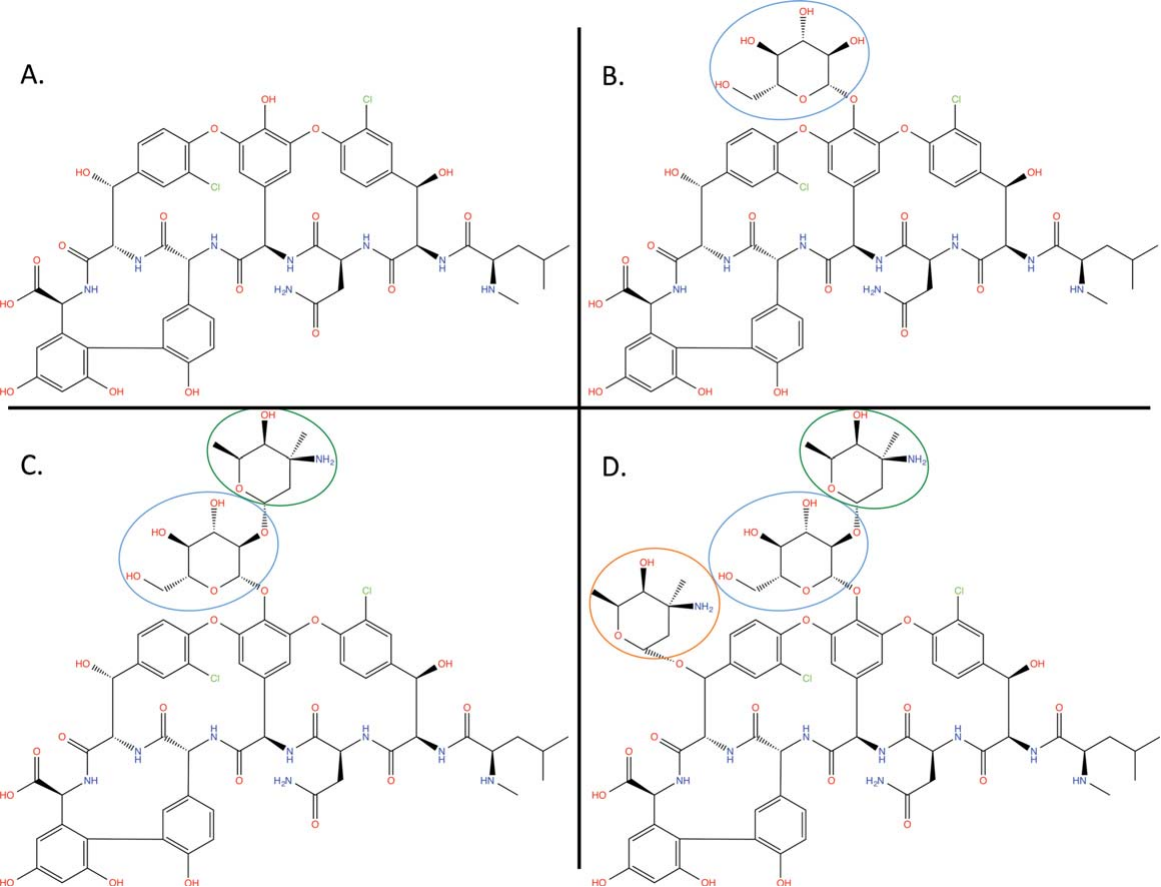
Chemical structures of four members of the vancomycin family of glycopeptide antibiotics. All these molecules have the same peptide core, and the different decorations in each of the molecules are circled using different colors. (A) Vancomycin aglycon has no sugars linked to it (PDB entry 1ghg).^15^ (B) Desvancosaminyl vancomycin is an intermediate in the vancomycin-biosynthesis pathway. It has only one saccharide linked to the peptide core (PDB entry 1rrv).^16^ (C) Vancomycin has a disaccharide decorating the peptide core (PDB entry 1aa5).^17^ (D) Chloroorienticin A has a disaccharide and a monosaccharide decorating the core (PDB entry 1gac).^18^

In the BIRD, resource-related PRD molecules are grouped together into families (FAM entries), based on chemical similarity. For naturally derived peptide-like molecules and their derivatives, the PRD molecules are assigned to families based on conservation of the core polymer sequence or the presence of signature sequence motifs. For example, several vancomycin-related glycopeptide antibiotics are grouped into one family (FAM_000087), in which all members have a conserved peptide core sequence but are decorated with a variety of polymeric or nonpolymeric groups. Selected members of this family are shown in Figure 5. For designed peptide-like inhibitors, the classification is first based on the conservation of the biologically active residue(s) or sequence motif(s) critical for its binding and/or function, followed by sequence conservation in the rest of the polymer/subcomponent sequence. The FAM entries include descriptions of various properties of member PRD molecules (such as synonyms, references to other resources and databases with information about them) and annotations (such as functions, mechanism of action, and pharmacological action) (TableII). The FAM entries also include identifiers of related molecules present in the Cambridge Structural Database (CSD)^19^ as well as family-specific literature references. The current set of FAM files is available for download from the wwPDB ftp server (http://ftp://ftp.wwpdb.org/pub/pdb/data/bird/family/) and its mirrors are at the wwPDB partner sites.

Peptide-like molecules in many of the families share similar chemistries at their business end but may have significantly different sequences elsewhere. While these molecules interact with their target macromolecules in the same way, sequence-based comparisons would not be able to cluster them together. An additional level of classification, called family groups (FGRs), was developed to organize families based on the mechanism of interaction of the peptide-like molecules with their target molecules. The chemistry of the peptide-like molecules, both before and after binding to their target macromolecules, is considered for this classification. The FGRs are assigned unique identifiers and the relationship of FGR IDs and FAM IDs is listed in an index file, available for download along with the FAM files. While many of the FGRs contain a single family (e.g., FGR_000079 at present only contains the vancomycin-like family FAM_000087), there are several FGRs with multiple families, each containing one or more PRD members (such as various chloromethylketone inhibitor families grouped into FGR_000008). A single family (FAM) can belong to multiple FGRs based on different criteria, such as pre-and post-binding chemistries. FGRs containing three or more released PRD entries (assigned to either a single or multiple families) were included in the index file and the corresponding FAM files were released. Currently, over 1100 released PDB entries have one or more instances of ∼660 different released PRD entries. Of these, ∼580 PRD entries have been assigned to 193 released family entries and classified in 64 FGRs. Analysis and classification of the family and FGRs is ongoing. The FAM and FGR classifications will be annually reviewed and updated by the wwPDB and released for general use.

## DISCUSSION

**Figure 6:**
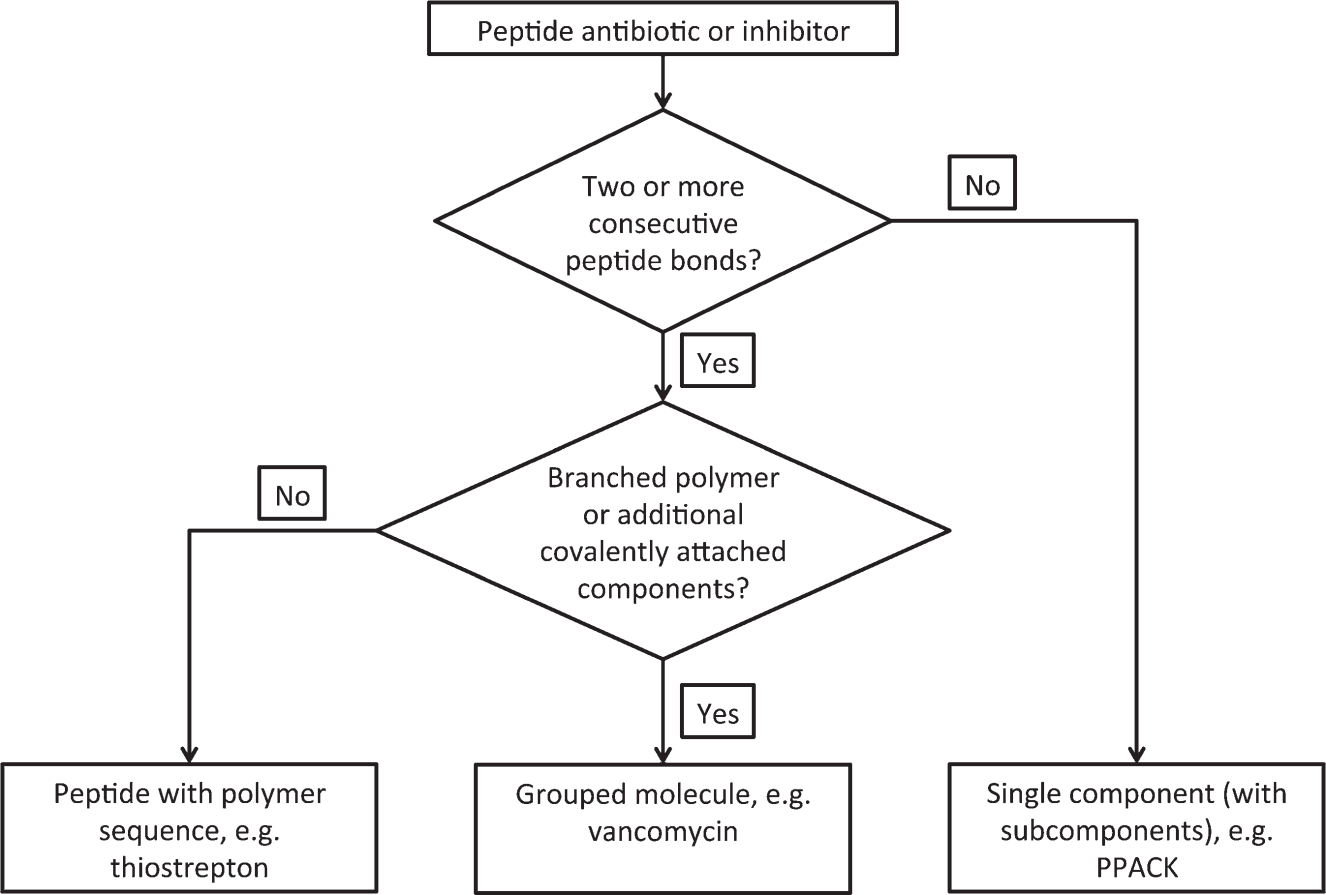
A flow-chart showing the logic used for deciding the representation of peptide-like inhibitor and antibiotic molecules in the PDB.

The BIRD resource is a by-product of the remediation effort. It informs and assists in current wwPDB procedures for annotation of peptide-like inhibitor and antibiotic molecules. The remediation of the peptide-like inhibitor and antibiotic molecules has improved the representation of these molecules in the PDB archive, and facilitated the development of new guidelines, tools, and procedures for annotation of these molecules. Using these new tools, candidate peptide-like inhibitor and antibiotic molecules can be automatically compared against the CCD and BIRD resources based on two-dimensional, 3D, and sequence matches. Annotators can review the matches and decide how to properly annotate the molecules. If a molecule does not match any existing CCD or PRD entry, its composition and/or sequence is used for deciding its representation. Based on the experience gained during the archive-wide remediation, guidelines were developed for deciding the representation of peptide-like molecules. A flowchart of the decision process is shown in Figure 6. New tools are now available to “chop” a large single ligand into its monomeric components or subcomponents, or to merge several residues into a single component while retaining the subcomponent sequence. The BIRD resource also provides a reference for validating the composition and connectivity of these molecules. In summary, new annotation tools, guidelines, and the BIRD resource enable consistent annotation of peptide-like inhibitor and antibiotics in the PDB, regardless of their representation in the initial deposition.

Of the PDB entries deposited and processed between September 2012 and August 2013, ∼125 PDB entries contained one or more peptide-like inhibitor or antibiotic molecules. This corresponds to a little over 1% of all deposited PDB entries during this period. Of the ∼100 distinct PRD molecules encountered in these entries, 87 were new additions to the BIRD resource. As more peptide-like molecules are added to the BIRD resource, new sequence patterns and signature chemical groups for peptide-like inhibitors and antibiotics may emerge. Systematic queries of the PDB archive for instances of these newly identified sequences and chemical groups may identify additional peptide-like molecules that were missed during the remediation. These molecules will be annotated and included in the BIRD resource as and when they are identified.

An important application of the BIRD resource is to facilitate and enhance various queries of the contents of the PDB. The chemical descriptions in PRD and FAM entries can be used to either search for a whole molecule or for signature chemical groups (such as thiazole/thiazoline groups). This form of query is particularly important for molecules comprised of several polymeric and nonpolymeric components and for chemical groups assembled from different parts of a polymer or from combinations of polymer and nonpolymer components. Before remediation and creation of the BIRD resource, such queries and analyses were not possible.

The remediation of peptide-like inhibitors and antibiotics has led to the creation of novel representations such as the subcomponent sequence and group. These representations will be used in future remediation activities covering other classes of molecules such as carbohydrates and lipids. While the BIRD resource currently covers only peptide-like molecules, it can be extended to include other biologically interesting molecules and information about them from other databases and resources.

## MATERIALS AND METHODS

### Dictionaries and Files

The master format for the entries in the PDB archive is PDBx/mmCIF^13^ (http://mmcif.pdb.org/dictionaries/ascii/mmcif_pdbx.dic), which presents all information in specific data categories defined in the expanded PDB Exchange Dictionary based on the mmCIF dictionary.^20^ The legacy record-oriented PDB format can be mapped to PDBx (http://mmcif.pdb.org/dictionaries/pdb-correspondence/pdb-mapping-v33.html.).

Complete chemical descriptions of all residues encountered in PDB entries are available in the CCD^3^ (http://ftp://ftp.wwpdb.org/pub/pdb/data/monomers/components.cif.gz), which is also in PDBx format. In addition to standard and modified amino acids and nucleotides, the CCD includes chemical descriptions for small molecules such as ions, cofactors, drugs, inhibitors, and antibiotics. Components in the CCD are defined as complete and neutral molecules that include leaving atoms (atoms lost during polymerization or other chemical reactions) and missing atoms (atoms missing due to disorder in the first or representative entry in which the molecule was observed). Chemical components are reused throughout the archive for consistency and to facilitate comparison and analysis.

Biological macromolecules (such as proteins or nucleic acid polymers) are represented as a sequence of components (residues) with implied standard bonds linking them. Nonstandard linkages between residues (such as isopeptide linkages and disulfide bonds in proteins) are explicitly described in each PDB entry. During annotation, the geometry and stereochemistry of all components present in a PDB entry are checked and validated against existing components in the CCD.^3^ More recently, the geometry of components is also being validated against high-resolution experimentally determined data from the CSD^2^ using tools such as Mogul.^21^

### Identification of Peptide-Like Inhibitors and Antibiotics in the PDB

All PDB entries that were found to contain peptide-like inhibitor or antibiotic molecules, either in isolation or in complex with a target biological macromolecule, were included in the remediation. Various methods were used to identify these molecules as comprehensively as possible. All short (3–25 residue) peptide and peptide-like molecules in the PDB were identified and their functions were assessed based on compound name and keywords included in the PDB entry or in the literature. Text-based searches for names (such as vancomycin), chemical descriptions (or type, such as glycopeptide) and functions (or class, such as antibiotic) of known peptide-like inhibitor and antibiotic molecules helped identify many instances in the PDB. Recognition of specific chemical signatures that are unique to these molecules, such as a thiazole ring, or the presence of chemical components such as phenylglycine and compounds such as hemiacetals and hemiketals (formed as a result of covalent interactions between enzymes and inhibitors) also helped in the identification.

### Remediation of the Peptide-Like Inhibitors and Antibiotics

Each PDB entry containing a peptide-like inhibitor or antibiotic was reviewed to decide if its representation needed to be corrected. New chemical components were created for components that either did not exist in the dictionary or were incorrectly or inconsistently used in the polymer or subcomponent sequences. Existing single components for peptide-like inhibitors were updated with subcomponent sequences where appropriate, and the corresponding subcomponent atoms were grouped and annotated within that chemical component.

For each peptide-like inhibitor and antibiotic, PRD entries were created. All PDB entries that contain the PRD molecule were checked and necessary annotations were made. Both natural and designed peptide-like molecules with at least two consecutive peptide bonds were matched against sequence resources such as UniProt^22^ for gene products, and Norine^23^ or the literature for nonribosomal products. Any deviations from the reference sequences were recorded. If no suitable reference sequence could be identified, the sequence from the PDB entry itself was taken as the reference. The name, source organism, and linkage of components in the polymer were annotated just as for any other polymer in a PDB entry. For peptide-like molecules with fewer than two consecutive peptide bonds, the molecule name was derived from the CCD and its composition was presented as the subcomponent sequence (also derived from the CCD). Finally, for peptide-like antibiotics and inhibitors represented as grouped molecules, a list of the constituents for each instance of the grouped molecule was included along with an explicit description of the linkages between these constituents. All residues in the macromolecular target surrounding or interacting with the peptide-like molecule of interest were highlighted in the entry.

### Creating and Maintaining BIRD

The initial set of PRD entries was manually created for the remediated peptide-like inhibitor and antibiotic containing PDB entries, released in July 2011. Following this, PRD entries are being created at the time of annotation using new software and tools developed for this purpose.

Measures are in place to ensure that all instances of PRD molecules in the PDB are appropriately annotated. The entire PDB archive is regularly scanned for instances of known and released PRD molecules, to identify PDB entries that have the same representation (polymer sequence or chemical component ID) as in the PRD, but are not appropriately annotated. Once identified, these PDB entries are reviewed and corrected as necessary. In future, attempts will be made to identify and update any missed PRD instances that have representations different from that used in the PRD. Despite these efforts it may still not always be possible to systematically identify instances of known PRD molecules, for instance due to poor geometry or errors in chemistry. A new deposition system has been developed where depositors can identify and inform annotators about peptide-like inhibitors and antibiotics during deposition of the entry so that these molecules are appropriately annotated.

The families were manually classified and populated with various items of information and links to other resources and databases. The FGR assignment was also manually defined. The classifications, annotations and resource-links in the family entries and the FGRs will continue to be reviewed and updated annually to provide users with up-to-date PRD-specific and family-specific information.
